# Steganography in color images with random order of pixel selection and encrypted text message embedding

**DOI:** 10.7717/peerj-cs.380

**Published:** 2021-01-28

**Authors:** Krasimir Kordov, Stanimir Zhelezov

**Affiliations:** 1 Department of Computer Informatics, Faculty of Mathematics and Computer Science, Konstantin Preslavski University of Shumen, Shumen, Shumen, Bulgaria; 2 Department of Computer Systems and Technologies, Faculty of Mathematics and Computer Science, Konstantin Preslavsky University of Shumen, Shumen, Shumen, Bulgaria

**Keywords:** Steganography, Text encryption, Color images steganography, Least-significant bit steganography, Steganographic analysis

## Abstract

Information security is major concern in modern digital ages, and the outdated algorithms need to be replaced with new ones or to be improved. In this article a new approach for hiding secret text message in color images is presented, combining steganography and cryptography. The location and the order of the image pixels chosen for information embedding are randomly selected using chaotic pseudo-random generator. Encrypting the secret message before embedding is another level of security designed to misguide the attackers in case of analyzing for traces of steganography. Evaluating the proposed stegoalgorithm. The standard statistical and empirical tests are used for randomness tests, key-space analysis, key-sensitivity analysis, visual analysis, histogram analysis, peak signal-to-noise ratio analysis, chi-square analysis, etc. The obtained results are presented and explained in the present article.

## Introduction

Steganology is an ancient science that is becoming more and more widely used with the development of digital information.

It consists of two main areas: steganography and steganalysis. Steganography is an interdisciplinary applied science field (Cox et al., 2007; Stanev & Szczypiorski, 2016), a set of technical skills and art for ways to hide the fact of transmission (presence) of information. It is one of the most effective approaches to protecting important information by hiding it (data hiding). High-tech steganography summarizes the areas for hiding messages using communication and computer technology, nanotechnology and modern advances in sciences such as biology, chemistry and others (Wu et al., 2020; Koptyra & Ogiela, 2020; Abd-El-Atty et al., 2020).

Steganalysis has exactly the opposite task. It combines methods and technologies for detecting secret steganographic communications. Along with the beginning of the development of modern ways of hiding information, at the end of the 20th century research in the field of steganalysis (Johnson & Jajodia, 1998; Provos & Honeyman, 2001; Fridrich & Goljan, 2002) has begun. Steganalysis is divided into two main categories: blind and targeted (Zhelezov, 2016). Targeted steganalysis methods have been developed to detect data embedded with specific stegoalgorithms and they are very accurate against certain stegomethods. The blind analysis methods are based on algorithms that require prior “training” with a series of empty and filled containers. The most characteristic of both types of analysis is that their methods are based on statistical dependencies in the analyzed subjects (Nissar & Mir, 2002). Such a method is POV (Pairs of Values) as part of the chi-square analysis (Westfeld & Pfitzmann, 1999; Fridrich, Goljan & Du, 2001).

### Related work

One of the latest research which shows successful Optical Character Recognition (OCR) steganography technique with good results in steganalysis, is presented in (Chatterjee, Ghosal & Sarkar, 2020). Other example of resent steganographic research is described in (Pak et al., 2020), where the authors are using chaotic map for constructing a steganographic algorithm. Popular methods for image steganography are analyzed in Table 1.

**Table 1:** Survey of some recent stego research.

Reference	Marvel, Boncelet & Retter (1999)
Method	The method uses a fast compressed-domain embedding technique to facilitate on-the-fly
	compressed-domain public-key steganography
Notes	Low probability of detection and leaving an observer unaware that the hidden data exist.
	The method works with grayscale images
Reference	Satish et al. (2004)
Method	The method is based on a scheme of chaos based spread spectrum image steganography (CSSIS)
	involving the use of chaotic encryption and chaotic modulation in spread spectrum image
	steganography (SSIS)
Notes	A novel scheme of the use of chaos based encryption. Robustness is achieved by interleaving
	the message using a chaotic sequence
Reference	Baby et al. (2015)
Method	The method is based on hiding multiple color images into a single color image using the
	Discrete Wavelet Transform
Notes	DWT increases the payload of the steganographic process by data compression. The proposed
	approach provides a good PSNR and SSIM value which establish the robustness of this work
Reference	Amirulhaqi, Purboyo & Nugrahaeni (2017)
Method	The method is based on Spread Spectrum Steganography and the Vigenere Cipher embedding
	in gif images
Notes	The method consists of three processes, namely the spreading, modulation, and insertion of a GIF image to the message
Reference	Yadav & Dutta (2017)
Method	The method is based on spread spectrum image steganography with RSA message encryption.
Notes	High level of security. Spreading the message all over the pixels of the cover media using pseudo
	random generator that generates random locations of pixels in an image and embedding
	message with Least Significant Bit algorithm to make it highly indiscernible
Reference	Gaurav & Ghanekar (2018)
Method	The method presents a steganography algorithm based on local reference edge detection
	technique and exclusive disjunction in the sharp edge region compared to the uniform region
	of the image
Notes	This paper presents an improved steganography technique on the basis of HVS system with
	an improved XOR technique
Reference	Chatterjee, Ghosal & Sarkar (2020)
Method	The method is based on Optical Character Recognition (OCR) based Steganographic technique.
Notes	Advantages—indicating correct classification by the model and high PSNR values. The method
	works with grayscale images. Slow embedding process for large images
Reference	Pak et al. (2020)
Method	The method is based on an improved 1D chaotic system model
Notes	The conventional one-dimensional (1D) chaotic map has a simple structure, which is easy to
	implement and has a low computational cost. The algorithm shows a good performance against
	statistical analysis attacks. Conventional one-dimensional (1D) chaotic map has a drawback
	that the range of chaotic behaviors is narrow and the distribution of key sequence is uniform

The main task of the steganographic algorithm is to reduce the efficiency of such methods and thus to increase its reliability. For this purpose, it is necessary to choose a method of embedding that does not violate the statistical dependencies.

For this reason, a Spread Spectrum Steganography approach (Marvel, Boncelet & Retter, 1999; Satish et al., 2004) based on a pseudo-random sequence generator has been chosen in this article. Additional text encryption is applied for transforming the secret message into unreadable character sequence for increasing the level of security of the proposed steganographic algorithm. In this approach, the resulting pseudo-random sequences are used to determine the message embedding positions. This leads to preserving the statistical dependencies in the container. Another advantage of this approach is related to some types of targeted steganalysis. They extract the values of the smallest bits of the file sequentially and analyze them for repetitive sequences. With the embedding method proposed in the article this type of steganalysis is completely ineffective.

### Motivation and justification

The text messages and the digital images are the most used information carriers concerning the data flow in the Internet and mobile communications. There are thousand of chat applications designed for short text messages correspondence using different ways to secure the communications between the users. Variety of cryptographic algorithms are implemented in order to protect the transferred information. Unfortunately, some of the encryption methods have become outdated and the new ones are being invented to improve secure communication. Information security will always be a major concern that motivates the development of new secret methods for data distribution and real-time communication. Such method is proposed in this work by combining two general scientific areas: steganography and cryptography.

### An outline of the proposed work

Our main focus is to present a different approach for classic LSB Steganography in images using random order of pixel selection and embedding encrypted text message. In order to achieve our goal the proposed technique requires the following steps:

- constructing novel pseudo-random generator
- secure text encryption, using the constructed pseudo-random generator
- choosing random pixels (in chaotic order) from an image, using the constructed pseudo-random generator for embedding information
- using traditional LSB pixel’s color modification for hiding, leaving no traces of steganography.

## Pseudo-random Generator Based on Duffing Map and Circle Map

For random pixel selection we are using pseudo-random generator (PRG) described in this section. Pseudo-random generators (also called Pseudo-random number generators (PRNG)) are software realized and unlike True-random generators (TRG) are easy for implementation with significantly lower cost and time consumption. This is why they are often used in cryptographic and steganographic systems. Examples of PRGs can be found in (Kordov, 2014, 2015b). The requirement resistance of PRGs is to different types of attacks are discussed in this section.

### Duffing map description

**Figure 1:**
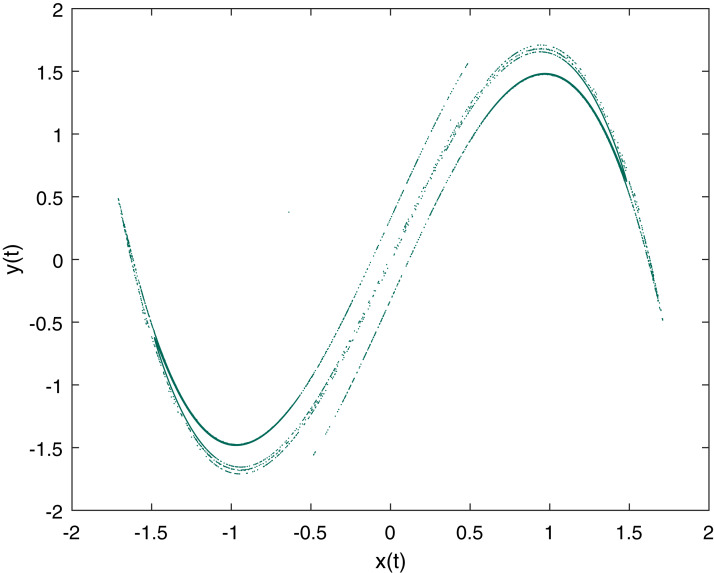
Duffing Map Plot with *a* = 2.75, *b* = 0.2, *x*_0_ = −0.63825 and *y*_0_ = 0.37713.

The Duffing map is well known two dimensional non-linear discrete-time dynamical system with chaotic behavior (Holmes & Moon, 1983) which is a discrete version of Duffing oscillator (Van Dooren, 1988). Duffing map is given by:

(1) $$\eqalign{&x_{t+1} = y_t \cr& y_{t+1} = -bx_t + ay_t - y_t^3,}$$

where *x*_*t*_ and *y*_*t*_ are double variables, calculated on every iteration, and *a* and *b* are fixed parameters of the Duffing map. For chaotic behavior the parameters are set to *a* = 2.75 and *b* = 0.2 (Hasan et al., 2017; Riaz et al., 2018). The initial test values we used for variables are *x*_0_ = −0.63825 and *y*_0_ = 0.37713 and Fig. 1 is a graphical representation of the Duffing map with the described values.

### Circle map description

**Figure 2:**
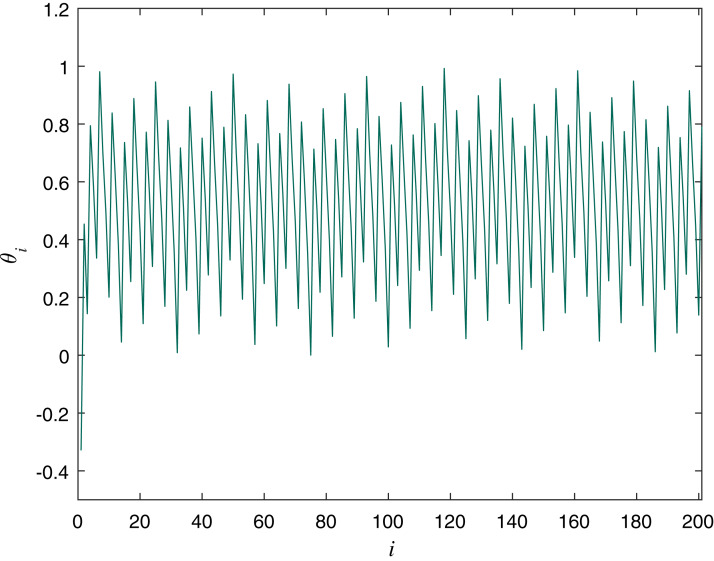
Circle Map Plot with Ω = 0.7128281828459045, *K* = 0.5 and θ_0_ = −0.329054.

The Circle map is explored for chaotic behavior in Shenker (1982) and DeGuzman & Kelso (1991). It has random-like properties and is suitable for constructing PRGs (Kordov, 2015a). The Standard circle map equation is:

(2) $${\rm \theta}_{i+1} = ({\rm \theta}_i + \Omega - \frac{K}{2\pi}\rm {sin(2\pi\theta_i)})\; \rm mod\; 1,$$

where θ is a double variable and Ω and *K* are the controlling parameters with values Ω = 0.7128281828459045, *K* = 0.5. The initial value for the variable we used for experiments in this article is θ_0_ = −0.329054. Figure 2 is a graphical representation of the Circle map with the described values.

### Random bit extraction process

The proposed bit extraction process is using Eqs. (1) and (2) and contains the following steps:

- The initial values of the constant parameters from Eqs. (1) and (2) are determined and the initial values of the double variables are set (described in the previous sections).
- For additional security, first *N* iterations from Eq. (1) and first *M* iterations from Eq. (2) are skipped. (we randomly chose *N* = *M* = 541).
- On every iteration of Eq. (1), *x*_*t*_ and *y*_*t*_ are used for calculation of additional double variable *p*_*t*_:

(3) $$\eqalign{&temp1_t = |integer(x_t \times 10^9)|mod\;2, \cr& temp2_t = |integer(y_t \times 10^9)|mod\;2, \cr& p_t = temp1 \; XOR \; temp2}$$

and θ_*i*_ from Eq. (2) is used for calculation of the variable *q*_*t*_:

(4) $$q_t = |integer({\rm \theta}_t \times 10^9)|mod\;2$$

- The produced random bit is obtained by performing XOR operation between the variables *p*_*t*_ and *q*_*t*_.
- The previous two steps are repeated until the necessary random binary sequence is reached.

### Key-sensitivity analysis

This test is performed to determine the behavior of the proposed PRG if there are changes in the secret key that is used to produce binary sequences. To test the key sensitivity very similar secret keys are used (described in Table 2) by changing a single digit in one of the initial double variables.

**Table 2:** Secret keys values.

Secret	Variable values		
Key	*x*_0_	*y*_0_	θ_0_
K1	−0.63825	0.37713	−0.329054
K2	−0.63824	0.37713	−0.329054
K3	−0.63826	0.37713	−0.329054
K4	−0.63825	0.37712	−0.329054
K5	−0.63825	0.37714	−0.329054
K6	−0.63825	0.37713	−0.329053
K7	−0.63825	0.37713	−0.329055

**Figure 3:**
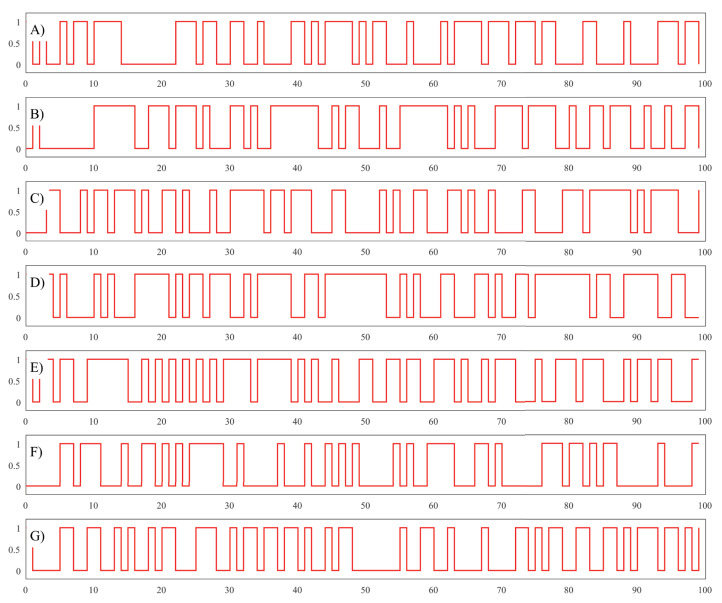
Circle Map Plot with Ω = 0.7128281828459045, *K* = 0.5 and θ_0_ = −0.329054. (A) Plot of binary sequence using secret key K1. (B) Plot of binary sequence using secret key K2. (C) Plot of binary sequence using secret key K2. (D) Plot of binary sequence using secret key K3. (E) Plot of binary sequence using secret key K4. (F) Plot of binary sequence using secret key K5. (G) Plot of binary sequence using secret key K6.

The results of the experiment are graphically presented in Fig. 3 and clearly show that the final binary sequence is different every time even if the secret keys are very similar. This means the proposed PRG is very sensitive to any changes in the initial conditions.

### Key-space analysis

The key-space includes the variety of possible values of the used variables in random bit generation. Equation (1) has two initial variables that can have different values (*x*_0_ and *y*_0_) and Eq. (2) has one variable −θ_0_. The parameters from the equations are constant so they cannot be part of the secret key. In addition to the secret key, the integer values of *N* and *M* also can have different values. Considering the floating point standard of IEEE for double variables (IEEE Computer Society, 2008) every double variable has precision about 10^−15^. Combining the three variables we have (10^15^)^3^ ≈ 2^149^ plus (2^32^)^2^ for the two integer variables and final about 2^213^ for key-space. The required key-space for resisting brute-force attacks is 2^100^ (Alvarez & Li, 2006) which means that the proposed PRG is secure enough. Kay-space comparison is presented in Table 3.

**Table 3:** Key-space comparison of PRGs.

Reference	Key-space
Kordov (2015a)	2^179^
Kordov (2014)	2^199^
Kordov (2015b)	2^199^
Proposed	2^213^

### Randomness evaluation

The most important property of a PRG is to produce random binary numbers. To evaluate the randomness 1 billion bits are generated and the sequence is tested with the most popular statistical test software packages.

#### NIST—random test

The first software for randomness evaluation is NIST—Statistical Test Suite (Bassham et al., 2010) and includes 17 base tests. The testing process is performed by dividing the tested sequence into 1,000 subsequences with length of 1,000,000 bits. All the NIST need to have *P*-values in the range [0,1) to be considered for successfully passed. The results for all tests are summarized in Table 4.

**Table 4:** NIST test suite results. The minimum pass rate for each statistical test with the exception of the random excursion (variant) test is approximately = 980 for a sample size = 1,000 binary sequences. The minimum pass rate for the random excursion (variant) test is approximately = 642 for a sample size = 657 binary sequences.

NIST test	*P*-value	Pass rate
Frequency	0.844641	991/1,000
Block-frequency	0.140453	993/1,000
Cumulative sums (Forward)	0.002820	993/1,000
Cumulative sums (Reverse)	0.723804	995/1,000
Runs	0.664168	991/1,000
Longest run of Ones	0.803720	996/1,000
Rank	0.473064	994/1,000
FFT	0.552383	991/1,000
Non-overlapping templates	0.513087	990/1,000
Overlapping templates	0.911413	996/1,000
Universal	0.494392	994/1,000
Approximate entropy	0.540204	989/1,000
Random-excursions	0.480341	650/657
Random-excursions Variant	0.531001	648/657
Serial 1	0.989786	993/1,000
Serial 2	0.413628	990/1,000
Linear complexity	0.574903	995/1,000

#### DIEHARD—ramdom test

The second test package is DIEHARD software (Marsaglia, 1995) and contains 19 test for randomness. The tests applied for the same bitstream of 1 billion bits generated by our PRG. The acceptable range again for calculated *P*-values is [0,1) for passing the individual tests. The results for all tests are summarized in Table 5.

**Table 5:** DIEHARD statistical test results.

DIEHARD test	*P*-value
Birthday spacings	0.3811619
Overlapping 5-permutation	0.7670145
Binary rank (31 × 31)	0.6561000
Binary rank (32 × 32)	0.4885290
Binary rank (6 × 8)	0.4461883
Bitstream	0.5432060
OPSO	0.6106608
OQSO	0.5272964
DNA	0.5160838
Stream count-the-ones	0.4152015
Byte count-the-ones	0.6557379
Parking lot	0.5087899
Minimum distance	0.2999120
D spheres	0.8421500
Squeeze	0.5968610
Overlapping sums	0.4620793
Runs up	0.6446245
Runs down	0.2258565
Craps	0.7826510

All the tests in Table 5 have *P*-values in range [0,1), indicating that all the tests for randomness evaluation are passed.

#### ENT—ramdom test

The ENT statistical test software (Walker, 2008) is the last package we used for randomness evaluation. The ENT software tests are: Entropy test, Optimum compression test, χ^2^ distribution test, Arithmetic mean value test, Monte Carlo π estimation test, and Serial correlation coefficient test. In Table 6 are presented the results for all tests from ENT software.

**Table 6:** ENT statistical test results.

ENT test	Result
Entropy	7.999999 bits per byte
Optimum compression	OC would reduce the size of this
	125,000,000 byte file by 0%
*χ*^2^ distribution	For 125,000,000 samples is 211.12,
	and randomly would exceed this
	value 97.92% of the times
Arithmetic mean value	127.4897 (127.5 = random)
Monte Carlo π estimation	3.142024754 (error 0.01%)
Serial correlation coefficient	−0.000085 (totally uncorrelated = 0.0)

## Steganography in Color Images with Random Pixel Selection

The proposed method combines the classical Least-significant bit (LSB) value replacement by choosing random positions of hiding in the image. The random order and the message encryption is performed by the proposed PRG.

### Message embedding algorithm

The process is performed by the following steps:

1. The text information is transformed to vector *V* of binary sequence using ASCII table values of the characters.

2. Control character sequence marking the end of the secret message is converted also in binary sequence and added to the vector *V*. (In our case we used “*#”).

3. The binary sequence in vector *V* is encrypted using XOR operation and random sequence produced by PRG with Secret Key 1. The result vector is *V*′.

4. The proposed PRG is used with the Secret Key 2 to produce two times 24 bits for selecting random position in an image with following rule:

(5) $$\eqalign{&x \; position = integer(24bits) \; mod \; image \; width, \cr& y \; position = integer(24bits) \; mod \; image \; height}$$

5. If a pixel with position (*x*,*y*) is used the previous step is repeated until unused pixel position is found.

6. LSB technique is used for embedding three bits from vector *V*′ into RED, GREEN and BLUE color values of the selected pixel.

7. Steps 4–6 are repeated until the sequence from vector *V*′ is embedded into the final stego image.

### Message extraction algorithm

The process is performed by the following steps:

1. The proposed PRG is used with the Secret Key 2 to produce two times 24 bits for selecting random position in the image with the following rule:

(6) $$\eqalign{&x \; position = integer(24bits) \; mod \; image \; width, \cr& y \; position = integer(24bits) \; mod \; image \; height}$$

2. If a pixel with position (*x*,*y*) is used the previous step is repeated until unused pixel position is found.

3. The LSB values from RED, GREEN and BLUE colors are copied into vector *V′*.

4. Every 8 bits are transformed into char value and every last two obtained characters are compared with the control sequence that marks the end of the message (“*#”).

5. Steps 1–4 are repeated until the control sequence is reached.

6. The binary sequence in vector *V′* is decrypted using XOR operation and random sequence produced by PRG with Secret Key 1. The result vector is *V′*.

7. Vector *V* is transformed from binary sequence into ASCII chars equivalent forming the original hidden text message.

## Experimental Setup

For our empirical experiments we used 2.40 GHz Intel Core i7-3630QM Dell Inspiron laptop with 8 GB RAM, x64 Windows 10 Pro operating system. The proposed method is realized using C++ programing language and the test images are personal photos taken within our university region. Sixteen color images are selected—8 with dimensions 256 × 256 and 8 with dimensions 512 × 512. MATLAB R2016a software is used for histogram plotting and image analysis and processing. The initial values used for PRG are *x*_0_ = −0.63825, *y*_0_ = 0.37713, θ_0_ = −0.329054 and for *N and M* − 541.

## Steganographic Analysis

In this section, the most used tests for steganographic analysis are included for testing the proposed stego algorithm. The color images are tested by embedding secret messages with different length. The messages are random only for the experiments and contain 100 letters (800 bits), 200 letters (1,600 bits), 300 letters (2,400 bits), 400 letters (3,200 bits), 500 letters (4,000 bits), 1,000 letters (8,000 bits), and 2,000 letters (16,000 bits). All the test in Supplemental File 1 and 2.

### Visual analysis

**Figure 4:**
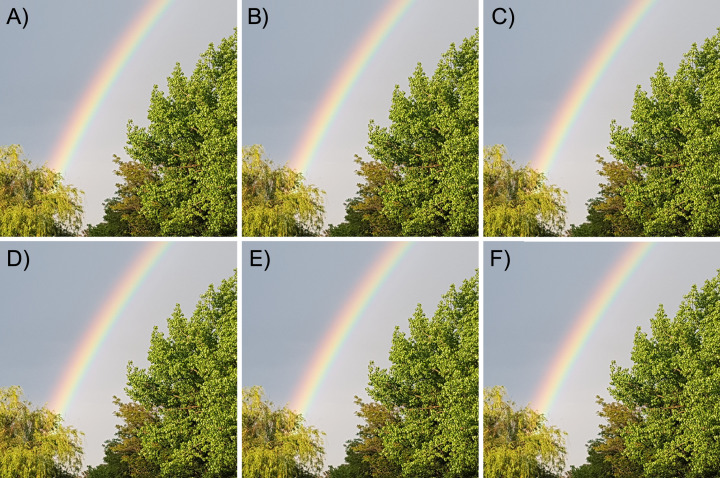
Visual comparison of a container image and corresponding stego images. (A) Container color image. (B) Stego image with 100 chars embedded. (C) Stego image with 200 chars embedded. (D) Stego image with 300 chars embedded. (E) Stego image with 400 chars embedded. (F) Stego image with 500 chars embedded. The photos were taken by the authors.

This is the most mandatory test for steganographic algorithm. A necessary requirement for any stego algorithm is to leave no visual traces of embedded secret messages or message container changes. Figure 4 shows one of the test images with its corresponding stego images with different lengths of embedded information.

**Figure 5:**
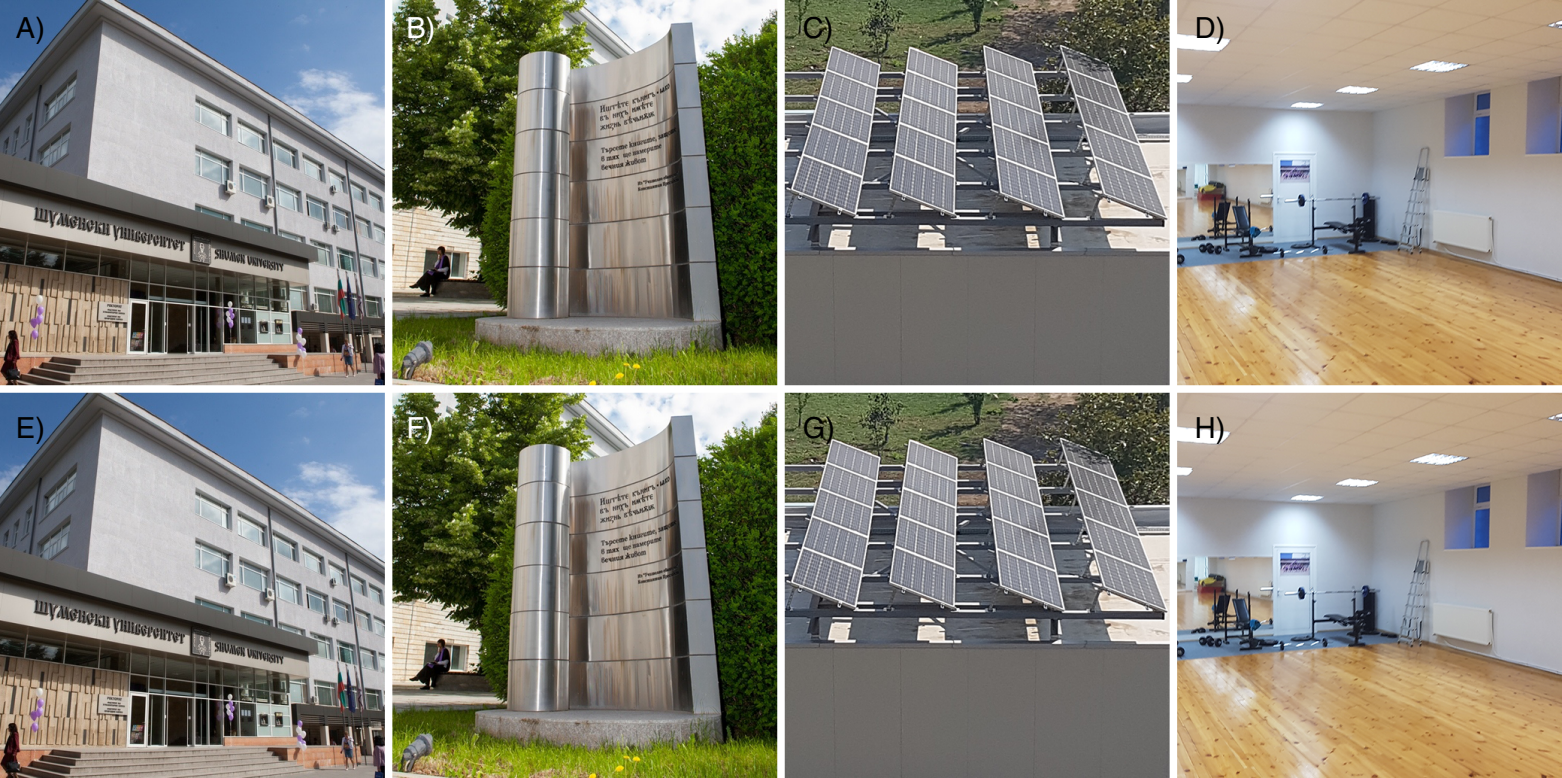
Additional examples of the proposed steganographic scheme. (A) Container image—Main corpus. (B) Container image—Monument. (C) Container image—Solarpanels. (D) Container image—Fitness. (E) Stego image—Main corpus. (F) Stego image—Monument. (G) Stego image—Solar panels. (H) Stego image—Fitness. The photos were taken by the authors.

Figure 4 clearly demonstrates that there are no visual differences between the images and no traces of hidden messages. More examples are presented in Fig. 5, to confirm that there are no visual trace of steganography in corresponding stego images.

### Histogram analysis

The image histograms are used for graphical representation of the tonal distribution of the red, green and blue colors. This experiment is designed to analyze if there are any changes in color distribution when the proposed steganographic method is applied.

**Figure 6:**
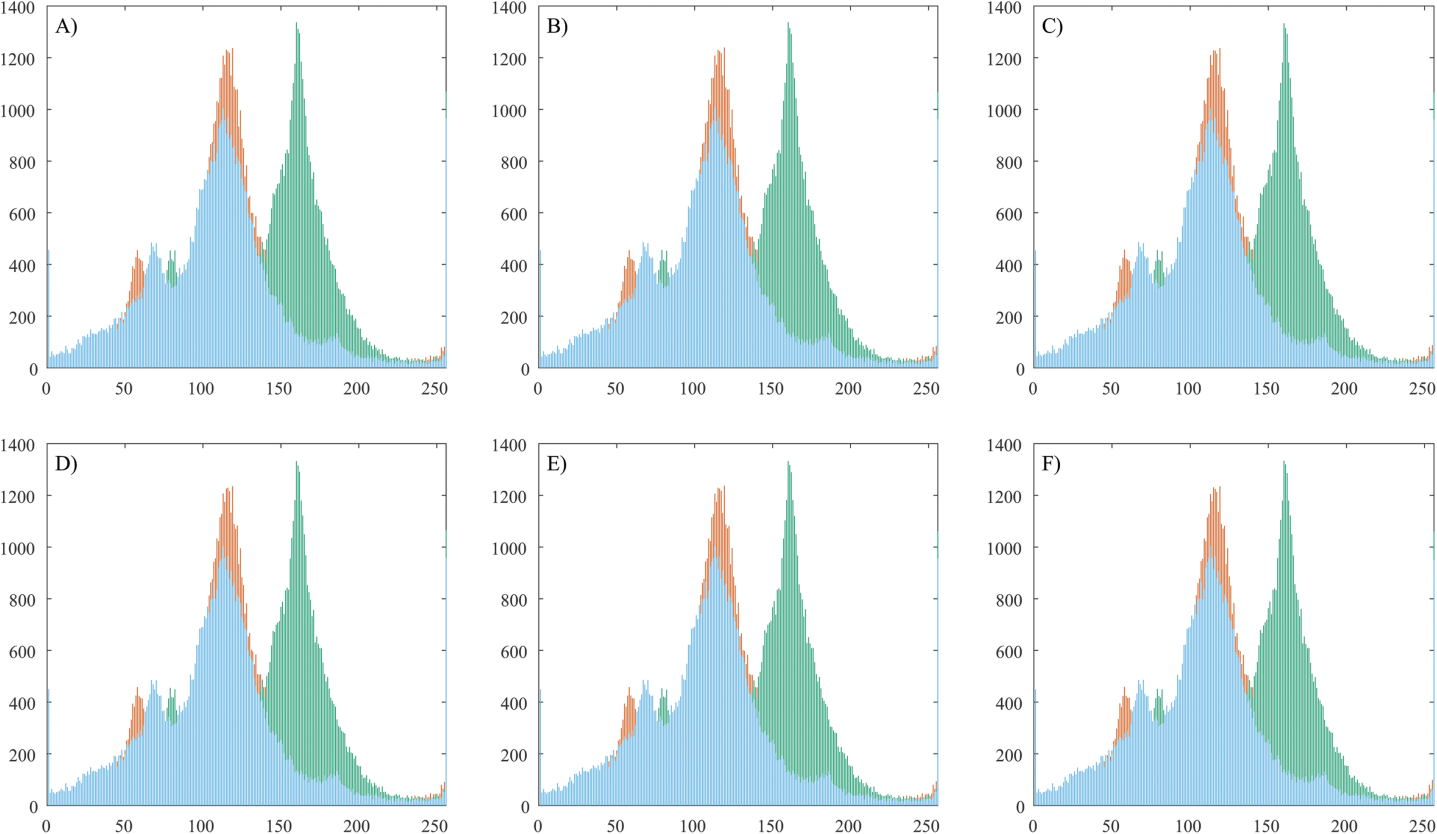
Histogram analysis of a plain image and corresponding stego images. (A) Container color image. (B) Stego image with 100 chars embedded. (C) Stego image with 200 chars embedded. (D) Stego image with 300 chars embedded. (E) Stego image with 400 chars embedded. (F) Stego image with 500 chars embedded.

Figure 6 shows the histograms of a test image with its corresponding stego images and Table 7 shows average pixel intensity values.

**Table 7:** Average pixel intensity comparison.

File name	Plain Image Red	Stego Image Red	Plain Image Green	Stego Image Green	Plain Image Blue	Stego Image Blue
f1-256x256	157.731	159.385	130.011	157.731	159.385	130.010
f2-256x256	152.790	127.875	102.467	152.790	127.875	102.466
f3-256x256	127.915	129.950	134.474	127.915	129.950	134.473
f4-256x256	111.816	115.017	86.290	111.816	115.017	86.290
f5-256x256	173.490	159.482	142.303	173.490	159.482	142.302
f6-256x256	114.214	114.245	111.757	114.214	114.245	111.757
f7-256x256	111.514	141.846	107.249	111.514	141.846	107.249
f8-256x256	120.503	117.243	108.933	120.503	117.243	108.933
f1-512x512	157.678	159.365	129.554	157.678	159.365	129.554
f2-512x512	152.831	127.877	102.465	152.831	127.877	102.465
f3-512x512	127.888	129.910	134.402	127.888	129.910	134.402
f4-512x512	111.793	115.004	86.060	111.793	115.004	86.060
f5-512x512	173.511	159.513	142.351	173.511	159.513	142.351
f6-512x512	114.465	114.475	111.955	114.465	114.475	111.954
f7-512x512	111.546	141.904	107.253	111.546	141.904	107.253
f8-512x512	120.562	117.298	108.986	120.562	117.298	108.986

The histogram attack method (Fridrich & Goljan, 2002) is historically the first statistical attack described in the resources. It is based on the fact that with LSB embedding, the even pixel values either remain unchanged (unmodified) or are being increased by 1, while the odd pixel values either remain unchanged or decrease. Thus, the values (2_*i*_, 2_*i*__+ 1_) form a pair of values (PoV), which are exchanged during embedding. This asymmetry in the embedding function can be used and a statistical test applied to confirm or deny that the even values follow the known distribution. The statistical steganalysis based on the Chi-square method is based on this. It makes a histogram (the frequency of occurrence of each color in the image) of the color distribution and based on it, pairs of adjacent values (PoV) are formed, differing in the youngest bit. Then a theoretical histogram of the color distribution is made, showing the expected distribution of the values in the presence of hidden information and pairs of adjacent values are formed again. The difference between the observed and expected occurrence frequencies for each pair is sought. In our case the observation of Fig. 6 shows that the tonal distribution is not changed when the secret messages are hidden in the plain image.

### Peak signal-to-noise ratio and structural similarity analysis

The Peak Signal-to-Noise Ratio (PSNR) measure the possible maximum power of the clean signal against the power of the noise signal. Poorly changing the pixel values of an image can lead to corruption of the image quality which may uncover a possible steganography. PSNR is calculated using the following equation:

(7) $${\rm PSNR} = 10{\rm log}_{10} {\frac{\rm MAX^2}{\rm MSE}} (dB),$$

where MAX is the maximum possible value of the pixel color. Considering that every pixel has 8 bits for red, green and blue color, we use the average value of the three values meaning MAX = 2^8^ − 1 = 255. MSE is the mean square error between the plain and stego images defined as:

(8) $${\rm MSE} = {\frac {1}\over{\rm NM}} \sum_{i=1}^N \sum_{j=1}^M (p_{x,y} - s_{x,y})^2$$

where *p*_*x*,*y*_ and *s*_*x*,*y*_ are the corresponding pixel values from the plain and stego images, respectively. Considering the color images have red, green and blue values for every pixel, the (*p*_*x*,*y*_ − *s*_*x*,*y*_)^2^ is calculated by:

(9) $${\frac {(rValue_{plain} {-}rValue_{stego})^2+(gValue_{plain}{-}Value_{stego})^2+(bValue_{plain}{-}bValue_{stego})^2}{3}}$$

The Structural Similarity (SSIM) is another method used in steganographic analysis proposed and described in Wang et al. (2004). The test is designed to determine the similarity between two images, in our case the similarity between plain and corresponding stego images. Values close to 1 are indicators for the best possible structural similarity between the compared images.

Part of the obtained values for MSE, PSNR and SSIM are shown in Table 8. The MSE and PSNR values are calculated for images with 100 chars (800 bits), 1,000 chars (8,000 bits), and 2,000 chars (16,000 bits) embedded. All the results are available in Supplemental File 3.

**Table 8:** The MSE, PSNR and SSIM values for images with 100 chars (800 bits), 1,000 chars (8,000 bits), and 2,000 chars (16,000 bits) embedded.

File name	Stego Image with 100 chars embbeded			Stego Image with 1,000 chars embbeded			Stego Image with 2,000 chars embbeded		
	MSE	PSNR	SSIM	MSE	PSNR	SSIM	MSE	PSNR	SSIM
f1-256x256	0.001587	76.12527	0.999985	0.016464	65.96539	0.999859	0.032562	63.00366	0.999723
f2-256x256	0.001480	76.42789	0.999986	0.015305	66.28259	0.999856	0.030289	63.31800	0.999715
f3-256x256	0.001541	76.25239	0.999993	0.016159	66.04664	0.999917	0.031754	63.11288	0.999845
f4-256x256	0.001511	76.33925	0.999996	0.015839	66.13363	0.999958	0.031494	63.14851	0.999921
f5-256x256	0.001434	76.56432	0.999987	0.015823	66.13782	0.999865	0.031296	63.17595	0.999732
f6-256x256	0.001556	76.20960	0.999992	0.015579	66.20535	0.999910	0.030823	63.24209	0.999825
f7-256x256	0.001633	76.00177	0.999993	0.015717	66.16723	0.999929	0.030640	63.26797	0.999862
f8-256x256	0.001526	76.29560	0.999993	0.015289	66.28693	0.999939	0.030563	63.27879	0.999878
f1-512x512	0.000404	82.06314	0.999996	0.003708	72.43954	0.999969	0.007530	69.36273	0.999936
f2-512x512	0.000366	82.49349	0.999996	0.003826	72.30319	0.999956	0.007805	69.20715	0.999910
f3-512x512	0.000397	82.14587	0.999997	0.003601	72.56648	0.999975	0.007263	69.51953	0.999949
f4-512x512	0.000408	82.02237	0.999998	0.003979	72.13336	0.999984	0.007813	69.20290	0.999969
f5-512x512	0.000381	82.31620	0.999996	0.003731	72.41281	0.999960	0.007599	69.32331	0.999917
f6-512x512	0.000347	82.72579	0.999998	0.003601	72.56648	0.999978	0.007313	69.48998	0.999955
f7-512x512	0.000404	82.06314	0.999997	0.004047	72.05905	0.999968	0.008007	69.09608	0.999938
f8-512x512	0.000370	82.44849	0.999998	0.003792	72.34234	0.999979	0.007710	69.26054	0.999958

Table 8 shows high values for PSNR (over 60 dB) meaning the stego algorithm do not destroy the image quality with considered minimum requirement of 20–30 dB for low quality. The obtained values for SSIM are close to the best possible value −1.

### Additional metrics analysis

Some researchers use different metrics for steganographic analysis of their methods. For evaluation of the proposed algorithm we performed additional experiments for the most used indicators—Average Difference (AD), Structural Content (SC), Normalized Cross-Correlation (NCC), Maximum Difference (MD), Laplacian Mean Squared Error (LMSE), Normalized Absolute Error (NAE), Image Quality Index (IQI). The best possible value for SC, NCC, MD and IQI is 1 and for AD, LMSE and NAE is 0. The results for our method are presented in Table 8. All the results are available in Supplemental File 3.

The obtained results in Table 9 show results close to the perfect values demonstrating the stability and efficiency of the proposed stegoalgorithm.

**Table 9:** The Average Difference (AD), Structural Content (SC), Normalized Cross-Correlation (NCC), Maximum Difference (MD), Laplacian Mean Squared Error (LMSE), Normalized Absolute Error (NAE), Image Quality Index (IQI).

File name	AD	SC	NCC	MD	LMSE	NAE	IQI
f1-256x256	0.000061	1.00000	0.999999	1.000000	0.000003	0.000010	1.000000
f2-256x256	0.000107	1.00000	1.000000	1.000000	0.000045	0.000011	1.000000
f3-256x256	0.000015	1.00000	0.999999	1.000000	0.000009	0.000012	1.000000
f4-256x256	−0.000107	0.99999	1.000001	1.000000	0.000008	0.000014	1.000000
f5-256x256	−0.000122	0.99999	1.000001	1.000000	0.000029	0.000009	1.000000
f6-256x256	0.000336	1.00000	0.999998	1.000000	0.000005	0.000014	1.000000
f7-256x256	0.000076	1.00000	0.999999	1.000000	0.000026	0.000013	1.000000
f8-256x256	0.000000	1.00000	1.000000	1.000000	0.000008	0.000013	1.000000
f1-512x512	0.000000	1.00000	1.000000	1.000000	0.000001	0.000003	1.000000
f2-512x512	0.000000	1.00000	1.000000	1.000000	0.000017	0.000003	1.000000
f3-512x512	0.000023	1.00000	1.000000	1.000000	0.000004	0.000003	1.000000
f4-512x512	−0.000019	1.00000	1.000000	1.000000	0.000004	0.000004	1.000000
f5-512x512	0.000008	1.00000	1.000000	1.000000	0.000024	0.000002	1.000000
f6-512x512	0.000034	1.00000	1.000000	1.000000	0.000003	0.000003	1.000000
f7-512x512	0.000038	1.00000	1.000000	1.000000	0.000078	0.000003	1.000000
f8-512x512	0.000072	1.00000	0.999999	1.000000	0.000006	0.000003	1.000000

### Comparison

In order to compare the proposed method with other image steganographic algorithms we use the presented metrics (where available) in related articles. The main metrics for defining the security and the reliability of the stegomethods are related to preserving the quality of the cover images and keeping the similarity with the stego images. For the image quality estimation the PSNR and MSE metrics are applied, and for the similarity of the cover and corresponding stego images—SSIM metric. The following Table 9 contains the most used metrics.

The test results in Table 10 show that the presented algorithm has satisfying statistical properties and provides better security level than compared methods.

**Table 10:** Average obtained values for PSNR, MSE and SSIM.

Reference	Average PSNR	Average MSE	Average SSIM
Proposed	72.302898	0.007316938	0.999953563
Chatterjee, Ghosal & Sarkar (2020)	51.1070	0.4990	0.9981
Yadav & Dutta (2017)	65.0345	0.0374	–
Amirulhaqi, Purboyo & Nugrahaeni (2017)	66.3124	0.1473	–
Baby et al. (2015)	55.4522	–	0.7764
Gaurav & Ghanekar (2018)	47.8039	1.4468	0.9989

### Chi-square analysis

In this article, steganalytic software based on the Chi-square method is used (available at http://www.guillermito2.net/stegano/tools/index.html). The software graphically shows the positions of the pixel values according to the image Chi-square value of the tested image. The red curve indicates the Chi-square values of the tested images and the green values represent the average value of the LSBs. If the green values are below the red curve the test didn’t pass successfully. Otherwise, it is assumed that the test was passed successfully, that is, there are no indications of a hidden message. For visual comparison we constructed a single screen shot image with six diagrams containing the results of the software.

**Figure 7:**
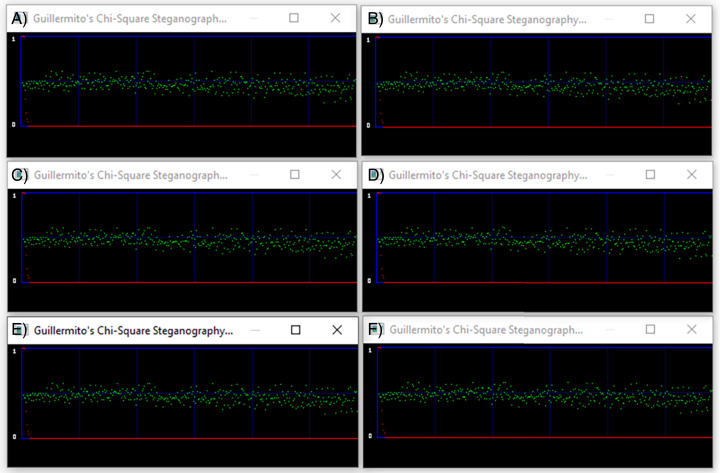
Chi-Square analysis of a container image and the corresponding stego images. (A) Container color image. (B) Stego image with 100 chars embedded. (C) Stego image with 200 chars embedded. (D) Stego image with 300 chars embedded. (E) Stego image with 400 chars embedded. (F) Stego image with 500 chars embedded.

Figure 7 demonstrates the results of our tests. The first is a diagram of the container and below are the corresponding stego files. The red curve is constantly at zero value leaving no green point under it. The Chi-square tests show that there is no trace of steganography in the stego files, indicated that the proposed algorithm can withstand against Chi-square attacks.

## Computational and Complexity Analysis

The proposed algorithm is tested with the conditions described in Experimental Setup Section. Concerning the complexity of our method, it is defined by the computations and iterations of the calculations for encryption and embedding operations. Considering the linear computation of every operation(random numbers generation, LSB modification etc.) do not affect the complexity, the theoretical complexity of the proposed scheme is θ(8 * *n*) equated to θ(*n*), where *n* defines the input data of the algorithm. The input data of the algorithm is the secret message for embedding which is processed as bit sequence (8 bits for a character). The image parameters (width and height) do not increase the time consumption, because the number of random selected pixels depends only from the length of the embedded secret message. However, the images size is related to the memory consumption.

The following Table 11 summarizes the results of our empirical experiment for embedding different size of secret text messages.

**Table 11:** Time complexity for secret text message encryption/decryption and embedding/extracting in color images.

File name	File dimensions	Length of the secret message				
		100 chars	500 chars	1,000 chars	1,500 chars	2,000 chars
		800 bits (s)	4,000 bits (s)	8,000 bits (s)	12,000 bits (s)	16,000 bits (s)
f1-256x256.bmp	256 × 256	0.001047	0.004402	0.008797	0.011602	0.016867
f2-256x256.bmp	256 × 256	0.000982	0.004679	0.009034	0.012969	0.017281
f3-256x256.bmp	256 × 256	0.001072	0.004439	0.009252	0.012871	0.017199
f4-256x256.bmp	256 × 256	0.001080	0.004546	0.008729	0.012998	0.017225
f5-256x256.bmp	256 × 256	0.001063	0.004473	0.008755	0.012997	0.017303
f6-256x256.bmp	256 × 256	0.001110	0.004423	0.008684	0.013166	0.016440
f7-256x256.bmp	256 × 256	0.001126	0.004425	0.009174	0.012913	0.017469
f8-256x256.bmp	256 × 256	0.001076	0.004401	0.008613	0.013002	0.016745
f1-512x512.bmp	512 × 512	0.001191	0.004556	0.008669	0.012760	0.017055
f2-512x512.bmp	512 × 512	0.001162	0.004795	0.008789	0.012951	0.017146
f3-512x512.bmp	512 × 512	0.001203	0.004614	0.008765	0.012883	0.017187
f4-512x512.bmp	512 × 512	0.001181	0.004670	0.009005	0.012835	0.017069
f5-512x512.bmp	512 × 512	0.000949	0.004530	0.009063	0.013088	0.016204
f6-512x512.bmp	512 × 512	0.001173	0.004571	0.008818	0.012261	0.017133
f7-512x512.bmp	512 × 512	0.001159	0.004522	0.008727	0.012643	0.017098
f8-512x512.bmp	512 × 512	0.001123	0.004362	0.008808	0.011896	0.017253

The results in Table 11 show that the proposed method is very fast and the computational complexity depends entirely of the secret text length.

## Conclusions

In this manuscript a new method for steganography is presented. The base of the proposed algorithm is a PRG used for secret message encryption and random pixels selection for data embedding. Proving the level of security the PRG is statistically tested for randomness and key-sensitivity, and the key-space analysis defines a necessary level of brute-force attacks resistance with minimum requirement 2^100^ for key-space.

The steganographic algorithm is evaluated with visual analysis, file size comparison, histogram analysis and chi-square analysis and the results show that there are no traces of steganography when a secret message is hidden in the tested color images. The PSNR analysis indicates that the quality of the signal in stego images remains high, considered that the good quality of the signal is above 20 dB. Additional tests indicates high similarity between cover and the corresponding stego images for proving the security and the reliability of the proposed scheme. The presented method can be improved for real-time video communication with embedded data.

## Supplemental Information

10.7717/peerj-cs.380/supp-1 Supplemental Information 1 The first part of the test images. Container images and stego images with 100, 200, 300 and 400 chars embedded. The photos were taken by the authors.

10.7717/peerj-cs.380/supp-2 Supplemental Information 2 The second part of the test images. Stego images with 500, 1000, 1500 and 2000 chars embedded.

10.7717/peerj-cs.380/supp-3 Supplemental Information 3 Numerical data. All the metrics of steganographic analysis.

10.7717/peerj-cs.380/supp-4 Supplemental Information 4 The most important aspects of the source code. The C++ code contains the Pseudorandom generator initial values and a function for extracting bits, the encryption and decryption code and bits embedding code.

## Additional Information and Declarations

### Data Availability

The following information was supplied regarding data availability: The source code are available in the Supplemental Files.
